# Thyroid autoimmunity at onset of type 1 diabetes as a predictor of thyroid dysfunction: a thirty-years retrospective longitudinal study

**DOI:** 10.3389/fendo.2025.1699111

**Published:** 2025-11-03

**Authors:** Gemma Carreras, Lilian C. Mendoza, Cristina Colom, Mireia Tirado-Capistros, Helena Sardà, José Luis Sánchez-Quesada, Antonio Pérez

**Affiliations:** ^1^ Department of Pediatrics, Hospital Santa Creu i Sant Pau, Barcelona, Spain; ^2^ Department of Pediatrics, Obstetrics and Gynecology, Preventive Medicine and Public Health. Autonomous University of Barcelona, Barcelona, Spain; ^3^ Institut de Recerca Sant Pau (IR SANT PAU), Barcelona, Spain; ^4^ Department of Endocrinology and Nutrition, Hospital Santa Creu i Sant Pau, Barcelona, Spain; ^5^ Department of Medicine, Autonomous University of Barcelona, Barcelona, Spain; ^6^ CIBER Bioengineering, Biomaterials and Nanotechnology (CIBER-BBN), Zaragoza, Spain; ^7^ Department of Endocrinology and Nutrition, Hospital Dos de Maig, Barcelona, Spain; ^8^ Cardiovascular Biochemistry, Institut de Recerca Sant Pau (IR SANT PAU), Barcelona, Spain; ^9^ CIBER of Diabetes and Metabolic Diseases (CIBERDEM), Instituto de Salud Carlos III, Madrid, Spain

**Keywords:** type 1 diabetes mellitus, thyroid autoimmunity, thyroid peroxidase (TPO) antibodies, thyroid dysfunction, age-related screening strategy

## Abstract

**Background:**

Thyroid autoimmunity commonly coexists with type 1 diabetes due to shared autoimmune mechanisms, and early recognition of thyroid dysfunction is crucial for optimizing metabolic control. However, there is no consensus regarding the optimal screening strategy for detecting thyroid disease in patients with type 1 diabetes. This study aimed to determine the long-term predictive value of thyroid peroxidase antibodies (TPO-Abs) at the onset of type 1 diabetes for the development of thyroid dysfunction and to evaluate the influence of age at diabetes onset.

**Methods:**

We conducted a retrospective longitudinal study at a tertiary university hospital in Barcelona, Spain, including 160 Caucasian patients consecutively diagnosed with type 1 diabetes between 1987 and 1994. All participants were followed for at least 10 years (mean follow-up 30.6 ± 4.5 years). TPO-Abs were measured at diabetes onset, and thyroid function was periodically assessed throughout follow-up. The incidence of thyroid dysfunction was analyzed according to TPO-Ab status and age at diabetes onset (<18 vs ≥18 years).

**Results:**

At diabetes diagnosis, 21.9% of patients were TPO-Abs positive. Antibody positivity was a strong predictor of thyroid dysfunction, conferring an eightfold increased risk compared with antibody-negative patients (RR 8.1, 95% CI 4.79–13.69, p<0.001). During follow-up, thyroid dysfunction also developed in initially antibody-negative patients, particularly in those diagnosed before 18 years of age, whereas cases were rare among those diagnosed in adulthood. The relative risk of thyroid dysfunction associated with TPO-Abs at diabetes onset was substantially higher in adults compared with youth (RR 12.6, 95% CI 6.10–25.81 vs. RR 3.4, 95% CI 1.35–8.71).

**Conclusion:**

A rational screening strategy for thyroid disease in asymptomatic patients with type 1 diabetes should include measurement of TPO-Abs and thyrotropin at diagnosis, followed by annual thyrotropin assessment in antibody-positive individuals. In patients diagnosed with type 1 diabetes before 18 years of age who are initially antibody-negative, repeat screening every two years from puberty through adulthood is recommended.

## 1 Introduction

Thyroid dysfunction in general population has a prevalence ranging from 4.6 to 8.9 depending on studies (1, 2). Prevalence increases with age and is most frequent in females. Although screening in asymptomatic adults is recommended by some organization, there is little evidence to support this practice (3, 4).

Type 1 diabetes and autoimmune thyroid dysfunction are both autoimmune diseases, in which circulating auto antibodies appear prior to clinical onset of the disease. The coexistence of these two conditions is well established, reflecting their common autoimmune pathophysiology and overlapping genetic susceptibility (5, 6). Thyroid autoimmunity and thyroid dysfunction are significantly more prevalent in individuals with type 1 diabetes than in the general population. Approximately 20%–25% of patients with T1D have positive TPO-Abs (7), which confers a significant higher risk of thyroid dysfunction in both adults (risk ratio 7, 95% CI 3–13) and children (risk ratio 49, 95% CI 16–150) (8).

In addition, both untreated hypo- and hyperthyroidism have been associated with poor glycemic control (7, 9, 10).

Therefore, systematic screening for thyroid disease in asymptomatic patients with type 1 diabetes is recommended by most scientific societies (4, 10, 11). However, most evidence is derived from pediatric studies, while data in adults remain limited (8, 12–15).

In a previous report (16), we demonstrated that thyroid peroxidase antibodies (TPO-Abs) measured at the onset of type 1 diabetes had high sensitivity and, particularly, high negative predictive value for the development of thyroid dysfunction over 10 years of follow-up. Here, we report the 30-year predictive value of TPO-Abs in the same cohort and evaluate the effect of age at diabetes onset, with the aim of suggesting an evidence-based screening strategy.

## 2 Methods

### 2.1 Study design and patients

We conducted a retrospective longitudinal study in a cohort of patients with type 1 diabetes followed for a minimum of 10 years. Between September 1987 and January 1994, 204 Caucasian patients were consecutively diagnosed with type 1 diabetes at a tertiary university hospital in Barcelona, Spain, according to the diagnostic criteria established by the National Diabetes Data Group (NDDG,1979), which represented the international standards in force during that period (17). One patient with hypothyroidism diagnosed prior to diabetes diagnosis and 17 patients without thyroid autoimmunity determination at onset were excluded, as well as one patient later reclassified as having type 2 diabetes. In addition, 25 patients who transferred to other treatment centers before reaching 10 years of diabetes duration and from whom no subsequent data was available were excluded. All were TPO-Abs negative at diabetes onset, remained euthyroid at their last visit, and had similar age and sex distribution to those included in the study. Their mean follow-up was 4.1 ± 2.0 years (range, 0–8).

### 2.2 Ethics statement

This study utilized anonymized clinical and laboratory data from hospital records. The protocol was approved by the Clinical Research Ethics Committee of the Hospital de la Santa Creu i Sant Pau (IIBSP-PER-2024-110). All procedures complied with current national and international legislation regarding human research, good research practices, the 2013 Declaration of Helsinki, and European data protection regulations. Informed consent was obtained from patients currently followed at our center. Being a retrospective observational study, the research team obtained permission to collect data from medical records in cases where consent could not be obtained (e.g., deceased patients or those no longer followed at our hospital).

### 2.3 Assessments

Thyroid autoimmunity was assessed at diabetes onset measuring TPO-Abs by hemagglutination, the usual technique at that time, considered positive at a dilution of 1/100. Known positive and negative serum controls were systematically included in each run to assess assay validity and to exclude non-specific reactions. TPO-Abs determination was repeated at different time points in initially negative patients, with changes in methodology during the whole follow-up period. Thyrotropin (TSH) was measured using a commercial non-competitive immunoassay. During follow-up, TSH was assessed every 1–2 years in TPO-Abs positive patients, while those who were antibody negative were monitored less frequently, usually every 2–5 years. This schedule reflected the clinical practice of the time, in the absence of formal international guidelines in the late 1980s and early 1990s. Thyroid function test were obtained periodically throughout the study, although the intervals between the assessment were not strictly regular. Consequently, the exact timing of thyroid dysfunction onset could not always be determined. To avoid overestimating the time at risk, patients who remained euthyroid were censored at the date of their last recorded thyroid function test, ensuring that follow-up ended when thyroid status was last verified. This approach prevented extending observation beyond the period of active surveillance while maintaining comparability between patients. Thyroid dysfunction was diagnosed in the presence of abnormal serum TSH levels, with or without symptoms (normal range varied throughout the whole follow-up period). Thyroid dysfunction included both hypothyroidism and Graves’ disease, and was defined according to 1990s recommendations, using a sensitive TSH assay and free thyroid hormone measurements: hypothyroidism by elevated TSH with low (or normal, if subclinical) free T4, and thyrotoxicosis/Graves by suppressed TSH with elevated free T4 and/or free T3, supported when necessary by typical clinical features and diffusely increased radioactive iodine uptake. These definitions were consistent with the diagnostic standards utilized in clinical practice during the study period (18). Follow-up data were collected until September 2023.

The incidence of thyroid dysfunction at the end of the follow-up period was calculated as cases per 10,000 patient-years. Patients were stratified by the presence or absence of TPO-Abs at type 1 diabetes onset to analyze the influence of these antibodies on thyroid dysfunction development. They were also stratified by age at diabetes onset: <18 years (youth) and ≥18 years (adults), to analyze the influence of age.

### 2.4 Statistical analysis

Data were analyzed using SPSS version 29 for Windows (IBM Corp., Armonk, NY, USA). Clinical characteristics between patients with and without thyroid dysfunction, as well as between those diagnosed before and after 18 years of age, were compared using the chi-square or Fisher’s exact test for categorical variables, and the Student’s t-test or Mann-Whitney U test for continuous variables. To assess the predictive value of TPO-Abs at diabetes onset for thyroid dysfunction, a survival analysis was performed. We employed the Kaplan–Meier method to estimate survival functions and used the log-rank test to compare survival distributions between patients with or without TPO-Abs positivity at diabetes onset. Patient who did not develop thyroid dysfunction during follow-up were considered censored. Possible confounding factors, including sex, age at diabetes onset, and, when available, the presence of islet cell antibodies (ICA), were evaluated in an univariate analyses. Variables with a p-value <0.10 were subsequently entered into a multivariate Cox proportional hazards regression model to identify independent predictors of thyroid dysfunction. Additionally, the log-rank test was applied to evaluate the predictive value separately in patients diagnosed before and after 18 years of age. Data are expressed as mean ± SD or median (range) for continuous variables, and as percentages or rates for categorical variables. Statistical significance was established at p<0.05. As a sensitivity analysis, survival curves and log-rank tests were repeated after excluding patients who died during follow-up without developing thyroid dysfunction, to assess the potential influence of this competing event.

## 3 Results

### 3.1 Cohort characteristics and thyroid dysfunction by TPO-Abs status

A total of 160 patients (63% male) were included in the study. The mean age at diabetes diagnosis was 23.8 ± 9 years (range, 7–65), and the mean follow-up duration was 30.6 ± 4.5 years (range, 10–37). The mean duration of follow-up was 30.3 ± 5.9 years for antibody-positive and 30.7 ± 5.9 years for antibody-negative patients (p = 0.72). The overall prevalence of thyroid dysfunction was 21.9% (35/160), corresponding to a cumulative incidence of 71.5 cases per 10,000 patient-years.

Twenty-five patients were TPO-Abs positive at diabetes onset, and 21 of them (84%) developed thyroid dysfunction (20 hypothyroidism, 1 Graves’ disease), after a median follow-up of 5 years (range, 0–25), at a mean age of 35 ± 13 years (range, 15–72).

During follow-up, 18 patients initially TPO-Abs negative converted to positive after an average of 17.3 ± 5.9 years (range, 5–26). Thirteen of them (72%) developed thyroid dysfunction (11 hypothyroidism, 2 Graves’ disease), most of them (10/13) simultaneously with antibody detection.

Only one patient with persistently negative TPO-Abs developed thyroid dysfunction: a case of subclinical hypothyroidism in a 29-year-old woman, 10 years after diabetes onset, identified during evaluation of secondary amenorrhea.

**Figure 1:**
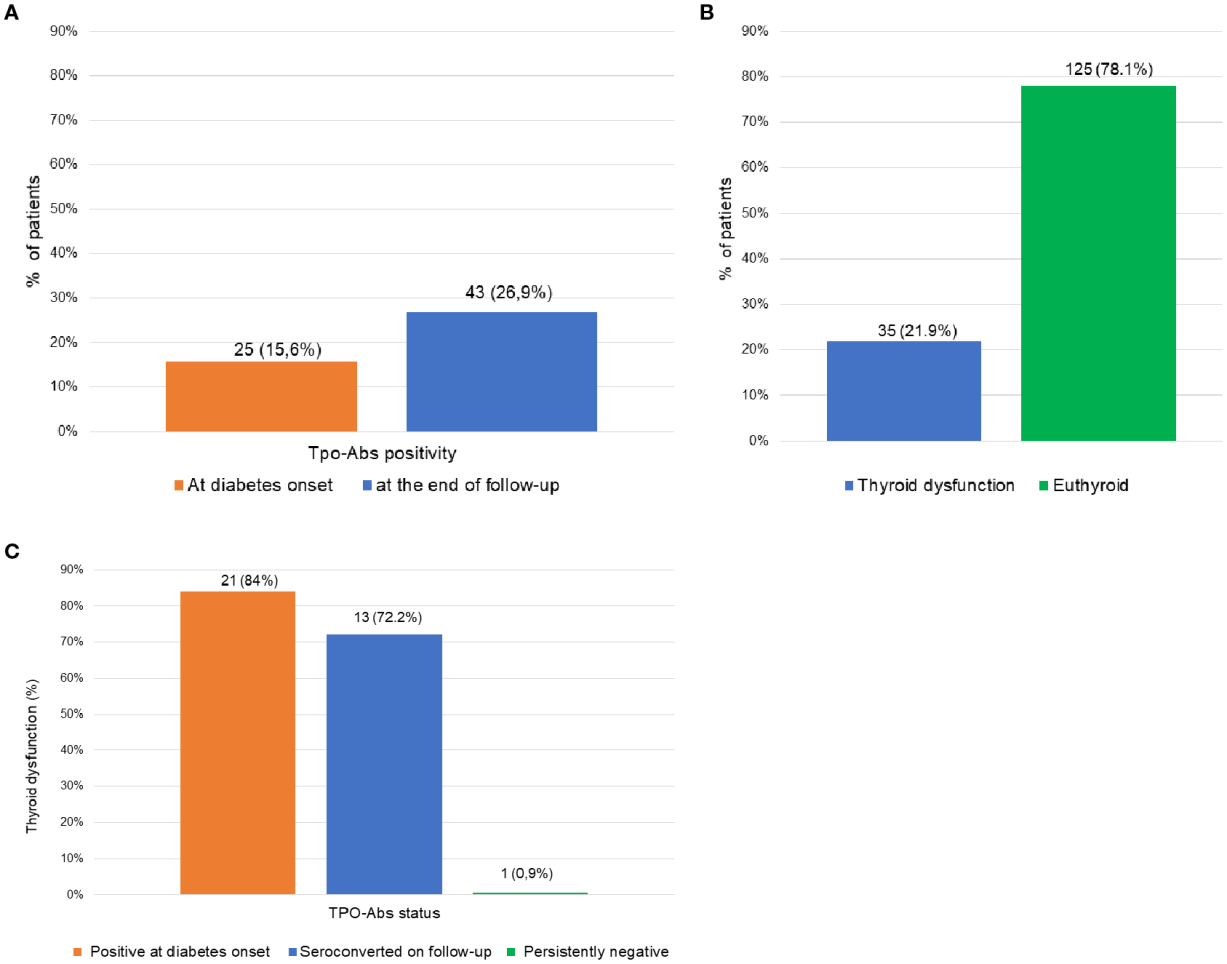
Prevalence and incidence of thyroid dysfunction and TPO-Abs positivity in the overall cohort. **(A)** Prevalence of TPO-Abs positivity at type 1 diabetes onset (n=25) and at the end of follow-up (n= 43), including seroconversions. **(B)** Cumulative incidence of thyroid dysfunction during follow-up (n=35). **(C)** Incidence of thyroid dysfunction stratified by TPO-Abs status: patients positive at diabetes onset (n=25), patients who seroconverted during follow-up (n=18), and those persistently negative (n= 117).

These data are illustrated in **Figure 1**, which presents the total prevalence and incidence of thyroid dysfunction and TPO-Abs in the overall cohort.

Baseline clinical characteristics of the overall cohort and according to age at diabetes onset (<18 vs ≥18 years) are presented in **Table 1**. **Table 2** summarizes thyroid dysfunction and TPO-Abs status in the overall cohort and by age at diabetes onset.

**Table 1:** Clinical characteristics of all patients and according to age at diabetes onset (<18 vs ≥18 years).

Variables	All patients (n=160)	Diabetes onset <18 years of age (n = 49)	Diabetes onset ≥18 years of age (n=111)	p value
Age, years	23.8 ± 9 (7–65)	14.4 ± 2.2 (7-17)	28.0 ± 7.6 (18-65)	<0.001
Sex, % male	63 (101/59)	55 (27/22)	67 (74/37)	ns
Follow-up, years	30.6 ± 4.5 (10-37)	30.6 ± 4.5 (12-37)	30.7 ± 4.5 (10-37)	ns
Age at the end of the study, years	54.4 ± 9.4 (28-82)	45.1 ± 4.8 (28-51)	58.6 ± 7.9 (33-82)	<0.001

Data are expressed as percentages and ratio or mean ± SD and range. p value refers to comparisons between youth and adults.

**Table 2:** Thyroid dysfunction and TPO-Abs positivity in all patients and according to age at diabetes onset (<18 vs ≥18 years).

Variables	All patients (n=160)	Diabetes onset <18 years of age (n = 49)	Diabetes onset ≥18 years of age (n=111)	p value
Prevalence of thyroid disfunction, %	21.9 (35/160)	22.4 (11/49)	21.6 (24/111)	ns
Cumulative incidence of thyroid dysfunction at the end of the study, cases/10.000 patients/year	71.5	73.4	71.1	ns
TPO-Abs positive at onset, %	15.6 (25/160)	14.3 (7/49)	16.2 (18/111)	ns
TPO-Abs seroconversion during follow-up, %	11.2 (18/160)	20.4 (10/49)	7.2 (8/111)	<0.05
Thyroid dysfunction associated with TPO-Abs seroconversion: - % of total thyroid disfunctions cases - cumulative incidence, cases per 10,000 patient-years	20 (13/35) 26.6	63.6 (7/11) 46.7	25 (6/24) 17.8	<0.05
Age at thyroid disfunction, years	36.6 ± 12.4 (15-72)	27.5 ± 7.9 (15-38)	40.8 ± 11.9 (19-72)	<0.01
Thyroid dysfunction with TPO-Abs (-), n	1	0	1	

Data are expressed as percentages and ratio, mean ± SD and range, or cases/10.000 patients/year.

p value refers to comparisons between youth and adults.

TPO-Abs positivity was more frequent in women (40.7% vs. 18.8% in men; p<0.01), as was thyroid dysfunction (33.9% vs. 14.9%, respectively; p<0.001).

TPO-Abs at diabetes onset were significantly associated with thyroid dysfunction (RR 8.1, 95% CI 4.79–13.69, p<0.001). Moreover, TPO-Abs at diabetes onset strongly predicted the subsequent development of thyroid dysfunction over the 30-year follow-up period (log-rank test: χ²=128.3; 95% CI 4.60–9.40; p<0.001). When the 25 patients who had transferred to other centers before completing ten years of follow-up were included as right-censored observations (log-rank χ² = 137.26; p < 0.001; 95% CI 4.6–9.4), the survival analysis results remained unchanged, confirming that their exclusion did not affect the main findings. In the Cox proportional hazards model, TPO antibody positivity at diagnosis remained independently associated with an increased risk of thyroid dysfunction (HR 211.9, 95% CI 28.1–1599.0; p<0.001). Neither sex nor age at diabetes onset was significantly associated with thyroid dysfunction in the multivariable model. Regarding other potential autoimmune markers, islet cell antibodies (ICA) were measured at type 1 diabetes diagnosis in 51.9% of patients (83/160), of whom 79.5% tested positive. ICA positivity showed no significant association with the subsequent development of thyroid dysfunction, in either univariate or multivariate analyses, and was therefore not retained in the final model.

### 3.2 Effect of age at diabetes onset on thyroid outcomes

As shown in **Table 2**, The prevalence of thyroid dysfunction did not differ significantly between patients with diabetes onset before or after 18 years of age. However, in adults, thyroid dysfunction was strongly associated with TPO-Abs positivity at diagnosis, whereas in youth, dysfunction was primarily linked to seroconversion during follow-up (p<0.05).

**Tables 3**–**5** show the accuracy of TPO-Abs at type 1 diabetes diagnosis to predict thyroid disfunction in the overall cohort and in subgroups stratified by age at diabetes onset. Although TPO-Abs predicted thyroid dysfunction in both groups, the relative risk was four times higher in adults (RR 12.6, 95% CI 6.10–25.81; **Table 4**) than in youth (RR 3.4, 95% CI 1.35–8.71; **Table 5**).

**Table 3:** Prevalence of thyroid dysfunction according to the presence of TPO-Abs at type 1 diabetes onset, and predictive values for future thyroid dysfunction development in the overall cohort.

	Thyroid dysfunction (n=35)	Euthyroid (n=125)	Total (n=160)	Predictive values and relative risk
TPO-Abs positive at diabetes onset	21	4	25 (15.6%)	PPV=84% (95% CI: 70–98)
TPO-Abs negative at diabetes onset	4	121	135 (85.7%)	NPV=89.6% (95% CI: 84–95)
Total	35 (21.9%)	125 (78.1%)	160 (100%)	
	Se= 60% (95% CI: 44–76)	Sp= 96.8% (95% CI: 94–100)		RR= 8.1 (95% CI: 4.8-13.7)

Se, sensitivity; Sp, specificity; PPV, positive predictive value; NPV, negative predictive value; RR, relative risk.

Reference standard: thyroid dysfunction confirmed by abnormal serum TSH, with or without symptoms.

Timeframe: mean follow-up 30.6 ± 4.5 years.

95% confidence intervals (CIs) calculated using the exact (Clopper–Pearson) method.

**Table 4:** Prevalence of thyroid dysfunction according to the presence of TPO-Abs at type 1 diabetes onset, and predictive values for future thyroid dysfunction development in adults-onset patients.

	Thyroid dysfunction (n=24)	Euthyroid (n=87)	Total (n=111)	Predictive values and relative risk
TPO-Abs positive at diabetes onset	17	1	18 (16.2%)	PPV=94,4% (95% CI: 72.7–99.9)
TPO-Abs negative at diabetes onset	7	86	93 (83.8%)	NPV=92,5% (95% CI: 85.1–96.9)
Total	24 (21.6%)	87 (78.4%)	111 (100%)	
	Se= 70.8% (95% CI: 48.9–87.4)	Sp= 98.9% (95% CI: 93.8–100.0)		RR=12.6 (95% CI: 6.10–25.81)

Se, sensitivity; Sp, specificity; PPV, positive predictive value; NPV, negative predictive value; RR, relative risk.

Reference standard: thyroid dysfunction confirmed by abnormal serum TSH, with or without symptoms.

Timeframe: mean follow-up 30.7 ± 4.5 years.

95% confidence intervals (CIs) calculated using the exact (Clopper–Pearson) method.

**Table 5:** Prevalence of thyroid dysfunction according to the presence of TPO-Abs at type 1 diabetes onset, and predictive values for future thyroid dysfunction development in patients diagnosed before 18 years of age.

	Thyroid dysfunction (n=11)	Euthyroid (n=38)	Total (n=49)	Predictive values and relative risk
TPO-Abs positive at diabetes onset	4	3	7 (14.3%)	PPV=57.1% (95% CI: 18.4–90.1)
TPO-Abs negative at diabetes onset	7	35	42 (85.7%)	NPV=83.3% (95% CI: 68.6 –93.0)
Total	11 (22.4%)	38 (77.6%)	49 (100%)	
	Se= 36.4% (95% CI: 10.9–69.2)	Sp= 92.1% (95% CI: 78.6–98.3)		RR= 3.4 (95% CI: 1.35–8.71)

Se, sensitivity; Sp, specificity; PPV, positive predictive value; NPV, negative predictive value; RR, relative risk.

Reference standard: thyroid dysfunction confirmed by abnormal serum TSH, with or without symptoms.

Timeframe: mean follow-up 30.6 ± 4.5 years.

95% confidence intervals (CIs) calculated using the exact (Clopper–Pearson) method.

**Figure 2:**
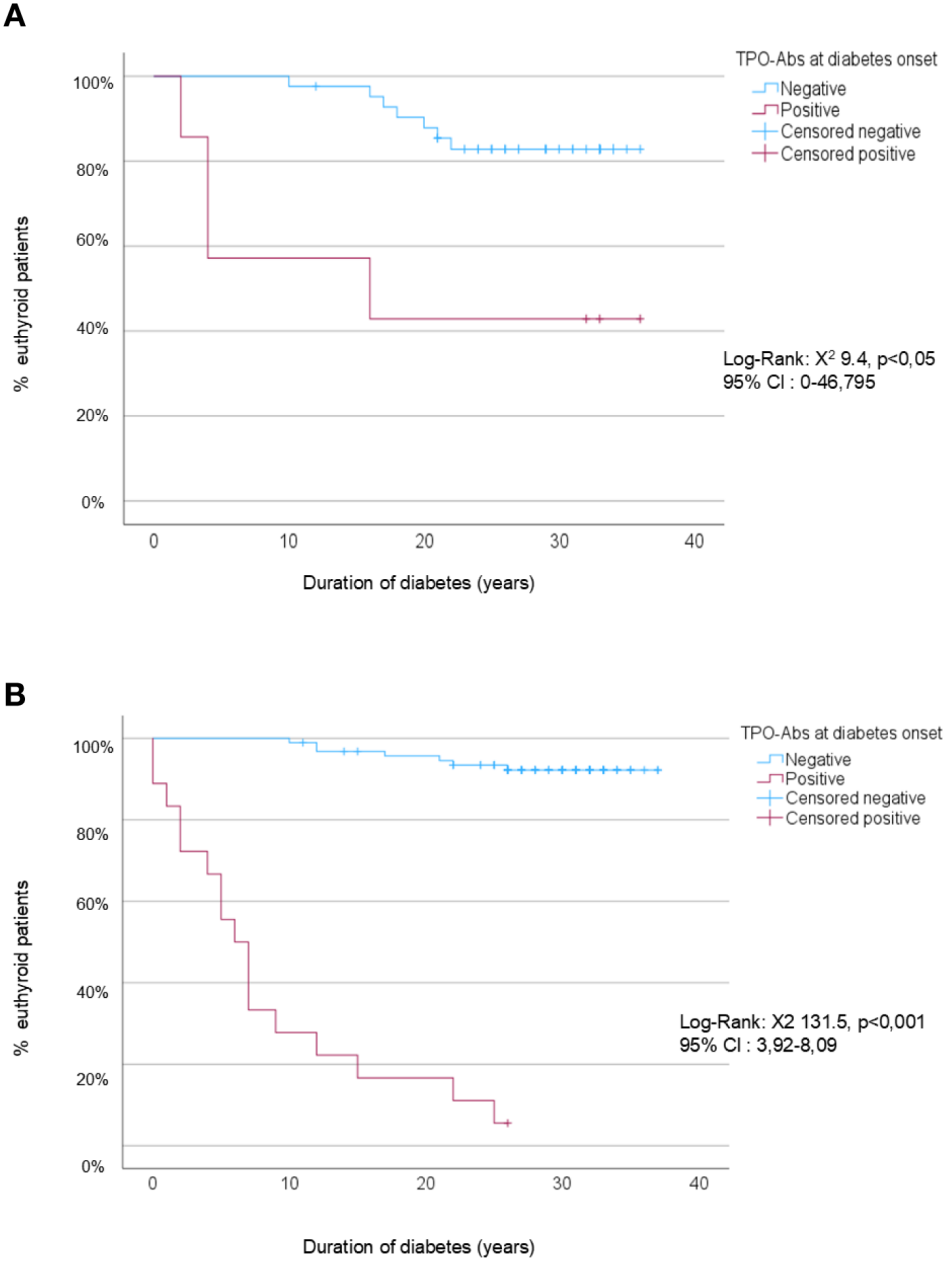
Kaplan-Meier survival analysis to predict thyroid dysfunction according to TPO status at diabetes onset. **(A)** Youth (<18 years at diabetes onset). **(B)** Adults (≥18 years at diabetes onset). • Censored negative: patients who were TPO-Ab (–) at diabetes onset and remained euthyroid at the end of the study or at their last follow-up visit. • Censored positive: patients who were TPO-Ab (+) at diabetes onset and remained euthyroid at the end of the study or at their last follow-up visit.

Survival curves also differed significantly by age (**Figure 2**). After 30.7 ± 4.5 years of follow-up, 92.5% of adults negative for TPO-Abs at diabetes onset remained euthyroid, compared with only 5.6% of those who were positive (log-rank: χ²=131.5; 95% CI 3.92–8.09; p<0.001). In contrast, among youth, 83% of initially negative patients remained euthyroid, compared with 42.8% of those positive at onset (log-rank: χ²=9.4; 95% CI 0–46.8; p<0.05). Excluding the seven patients who died during follow-up without developing thyroid dysfunction yielded results similar to the main analysis (log-rank χ² = 121.2; 95% CI, 4.6–9.4; p < 0.001), indicating the robustness of the findings.

## 4 Discussion

### 4.1 Main findings

This long-term longitudinal study confirms the high prevalence of thyroid dysfunction in patients with type 1 diabetes and supports systematic screening, in agreement with most scientific societies (4, 10, 11, 19). Although subjects with positive TPO-Abs at type 1 diabetes onset were eight times more likely to develop thyroid dysfunction than negative ones, the diagnostic accuracy was very high among subjects diagnosed in adulthood but lower in those diagnosed before the age of 18. Therefore, our study provides new insight supporting individualized screening strategies according to age at diabetes onset.

### 4.2 Thyroid autoimmunity as a determinant of thyroid dysfunction

Thyroid disease in type 1 diabetes is primarily autoimmune, with a significantly higher risk of thyroid dysfunction in subjects with positive thyroid antibodies (8). In fact, we only found one case of thyroid dysfunction in a persistently negative TPO-Abs patient. While some authors recommend annual TSH screening for all patients with type 1 diabetes (11, 20), others (8, 10, 14, 19, 21, 22) suggest testing both TSH and thyroid autoimmunity at diabetes diagnosis. This strategy can help identify patients who require closer monitoring for the development of thyroid disease, while reducing the frequency of testing in antibody negative individuals, potentially resulting in significant cost savings. Furthermore, determination of TPO-Abs alone may be sufficient, since they show greater sensitivity and predictive value than thyroglobulin antibodies (12, 14–17), contributing to further cost reduction. In our cohort, thyroglobulin autoantibodies were measured at diabetes diagnosis, but since they proved less sensitive than TPO-Abs in our previous report (16), we did not analyze them during follow-up.

### 4.3 Predictive value of TPO-Abs at diagnosis

In our previous report (16), we found that TPO-Abs at type 1 diabetes onset had high sensitivity and, particularly, a high negative predictive value for thyroid dysfunction within 10 years. Based on those findings, we proposed a strategy in which TPO-Abs should be determined at diabetes diagnosis, with annual TSH evaluation only in TPO-Abs positive patients. However, since the mean follow-up in that study was limited to 10 years, it remained unclear whether repeated antibody measurements would be required in the longer term, especially in younger patients, to detect possible seroconversion in the TPO-Abs negative group. In the present analysis, conducted in the same initial population, with extended follow-up period up to 30 years, we observed that some initially TPO-Abs negative patients seroconverted and developed thyroid dysfunction. These cases would have been missed using our formerly proposed screening strategy.

At type 1 diabetes diagnosis, TPO-Abs positivity is highly prevalent in both youth (<18 years) and adults (≥18 years), supporting universal screening at onset. Moreover, TPO-Abs positivity was associated with a high risk of thyroid dysfunction in the short term, with a median onset of 5 years, consistent with other studies (12, 13, 15). Therefore, annual TSH monitoring in these patients is justified and aligns with the recommendations of most scientific societies (10, 19, 21).

### 4.4 Screening implications in youth

Current evidence for repeating thyroid screening in asymptomatic patients who were TPO-Abs negative at type 1 diabetes diagnosis derived mainly from pediatric studies. In children and adolescents with type 1 diabetes, thyroid autoantibody prevalence increases with age, peaking during puberty, with age being a stronger predictor than diabetes duration (13–15). In children at genetic risk of type 1 diabetes, the peak incidence of thyroid autoimmunity occurs in early to mid-puberty, several years after the peak of islet autoantibodies around the age of 2 (23). Consequently, repeated screening in asymptomatic antibody-negative youth is warranted. Although the optimal frequency is unclear, repeating testing at least every two years from puberty or around age 12 appears reasonable (13). The International Society for Pediatric and Adolescent Diabetes (10) recommends repeating TSH every two years (grade E), while the American Diabetes Association suggests every 1–2 years (22), including antithyroid antibody testing and closer monitoring of patients who seroconvert.

In our study, 64% of thyroid dysfunction cases in youth occurred in patients who were initially TPO-Abs negative, emphasizing the importance of repeated screening in this age group. Despite earlier evidence indicating a high prevalence of thyroid autoimmunity in individuals with type 1 diabetes (13, 24), screening was performed at irregular intervals, reflecting the lack of specific ADA recommendations in the late 1980s and early 1990s. Formal guidance was only introduced later, initially specifically for children and adolescents, and many years later for adults (25, 26). As a result, the timing of seroconversion and thyroid dysfunction cannot be precisely determined. Nonetheless, since all cases were antibody-related and dysfunction did not always coincide with seroconversion, repeating both TSH and TPO-Abs, rather than TSH alone, may help identify high-risk patients who should be closely monitored and would be again cost-effective.

### 4.5 Screening implications in adults

In adults, most thyroid dysfunction cases were associated with TPO-Abs positivity at diabetes onset. Furthermore, 92.5% of antibody-negative adults remained euthyroid after more than 30 years of follow-up, with a mean age of 58 ± 8 years at the end of the study. The detection of five additional cases of subclinical hypothyroidism among 93 patients (3/29 women and 2/64 men) reflects a prevalence comparable to that of the general population at similar ages. Thus, repeated screening in asymptomatic antibody-negative adults does not appear justified.

In one of the few adult studies, Umpierrez et al. (20) recommended annual TSH determinations for all type 1 diabetes patients, since 5 of 18 hypothyroidism cases occurred in antibody-negative patients about 10 years after diagnosis. However, their cohort was small (n=58) and had a mean age at diabetes onset of only 19 ± 2 years, which in our classification would correspond to youth rather than adults.

### 4.6 Strengths and limitations

This study has some limitations. First, the measurement of TPO-Abs at diabetes diagnosis using a semiquantitative hemagglutination assay, reflecting the technology available at that time, but less sensitive than current immunoassays. The use of standardized techniques such as ELISA or CLIA would provide greater analytical accuracy, and future studies employing these methods could further validate our findings.

Second, the assessment of potential confounding factors included islet cell antibodies (ICA), which showed no significant association with thyroid dysfunction. However, data were available for only about half of the cohort, and, more importantly, antibodies against specific antigens were not determined at that time. Nevertheless, although glutamic acid decarboxylase antibodies (GADA) have been associated with thyroid autoimmunity in some studies, this association appears to occur primarily in young children (8, 14).

Third, thyroid function tests were not performed at strictly regular intervals, particularly during the early years of the cohort, so dysfunction onset timing could not always be determined. However, censoring at the last available thyroid test reduce the risk of time related bias.

Fourth, 25 patients transferred to other centers before completing ten years of follow-up were excluded. However, a sensitivity analysis treating them as right-censored observations showed that their exclusion did not influence the findings.

Finally, the retrospective observational design limits causal inference and precludes the use of competing-risk models. Nevertheless, mortality was low and evenly distributed between groups, and sensitivity analyses excluding deceased patients confirmed that deaths had minimal impact on the observed associations.

Despite these limitations, the exceptionally long and consistent follow-up provides robust, and clinically relevant evidence supporting an age-tailored approach to thyroid screening in type 1 diabetes.

### 4.7 Proposed screening strategy

Based on our findings, we propose an age-tailored screening strategy for asymptomatic thyroid disease in type 1 diabetes. TSH and TPO-Abs should be measured at diabetes diagnosis in all patients. Annual TSH testing should be limited to TPO-Abs positive individuals. In patients diagnosed before 18 years of age who are antibody-negative at onset, repeated screening with both TSH and TPO-Abs is advisable, at least every two years from puberty through adulthood. In asymptomatic antibody-negative adults, however, no further routine screening beyond diagnosis appears necessary. This targeted approach improves early detection in youth while minimizing unnecessary testing in adults, addressing a key gap in current guidelines, which are primarily focused on pediatric populations.

## Data Availability

The raw data supporting the conclusions of this article will be made available by the authors, without undue reservation.
