# Supplementary material for: Thyroid autoimmunity at onset of type 1 diabetes as a predictor of thyroid dysfunction: a thirty-years retrospective longitudinal study
Source: Front Endocrinol (Lausanne). 2025 Nov 3;16:1699111. doi: 10.3389/fendo.2025.1699111 (PMC12620207; doi:10.3389/fendo.2025.1699111)

Kaplan-Meier : PATIENTS<18 YEARS AT DABETES ONSET

Notes

Output Created		29-SEP-2025 14:03:23
Comments		
Input	Data	\\dspau.santpau.es\w\gcarreras\info\Escriptori\TIROIDES\listado AC 30-11-2023 angles.sav
	Active Dataset	DataSet1
	Filter	(seguimen >= 10) AND (edaddx < 18) (FILTER)
	Weight	<none>
	Split File	<none>
	N of Rows in Working Data File	49
Missing Value Handling	Definition of Missing	User-defined missing values are treated as missing.
	Cases Used	Statistics are based on all cases with valid data for all variables in the analysis.
Syntax		KM Supervivència BY tpoqbis<br>/STATUS=disftir(1)<br>/PRINT TABLE MEAN<br>/PLOT SURVIVAL<br>/TEST LOGRANK<br>/COMPARE OVERALL POOLED.
Resources	Processor Time	00:00:00,27
	Elapsed Time	00:00:00,25

Case Processing Summary

TPO at diagnosis +/-	Total N	N of Events	Censored N	Censored Percent
Negative at onset	42	7	35	83,3%
Positive at onset	7	4	3	42,9%
Overall	49	11	38	77,6%

Survival Table

TPO at diagnosis +/-		Time	Status	Cumulative Proportion Surviving at the Time: Estimate	Cumulative Proportion Surviving at the Time: Std. Error
Negative at onset	1	10,000	si	,976	,024
	2	12,000	no	.	.

Survival Table

TPO at diagnosis +/-		N of Cumulative Events	N of Remaining Cases
Negative at onset	1	1	41
	2	1	40

Survival Table

TPO at diagnosis +/-		Time	Status	Cumulative Proportion Surviving at the Time	
				Estimate	Std. Error
	3	16,000	si	,952	,033
	4	17,000	si	,927	,040
	5	18,000	si	,903	,046
	6	20,000	si	,879	,051
	7	21,000	si	,854	,055
	8	21,000	no	.	.
	9	21,000	no	.	.
	10	22,000	si	,828	,059
	11	23,000	no	.	.
	12	24,000	no	.	.
	13	24,000	no	.	.
	14	25,000	no	.	.
	15	25,000	no	.	.
	16	26,000	no	.	.
	17	26,000	no	.	.
	18	27,000	no	.	.
	19	27,000	no	.	.
	20	29,000	no	.	.
	21	29,000	no	.	.
	22	29,000	no	.	.
	23	30,000	no	.	.
	24	31,000	no	.	.
	25	31,000	no	.	.
	26	32,000	no	.	.
	27	33,000	no	.	.
	28	33,000	no	.	.
	29	33,000	no	.	.
	30	33,000	no	.	.
	31	33,000	no	.	.
	32	33,000	no	.	.
	33	33,000	no	.	.
	34	33,000	no	.	.
	35	34,000	no	.	.
	36	34,000	no	.	.
	37	34,000	no	.	.
	38	34,000	no	.	.
	39	34,000	no	.	.
	40	34,000	no	.	.
	41	35,000	no	.	.
	42	36,000	no	.	.

Survival Table

TPO at diagnosis +/-	N of Cumulative Events	N of Remaining Cases
3	2	39
4	3	38
5	4	37
6	5	36
7	6	35
8	6	34
9	6	33
10	7	32
11	7	31
12	7	30
13	7	29
14	7	28
15	7	27
16	7	26
17	7	25
18	7	24
19	7	23
20	7	22
21	7	21
22	7	20
23	7	19
24	7	18
25	7	17
26	7	16
27	7	15
28	7	14
29	7	13
30	7	12
31	7	11
32	7	10
33	7	9
34	7	8
35	7	7
36	7	6
37	7	5
38	7	4
39	7	3
40	7	2
41	7	1
42	7	0

Survival Table

TPO at diagnosis +/-		Time	Status	Cumulative Proportion Surviving at the Time: Estimate	Cumulative Proportion Surviving at the Time: Std. Error
Positive at onset	1	2,000	si	,857	,132
	2	4,000	si	.	.
	3	4,000	si	,571	,187
	4	16,000	si	,429	,187
	5	32,000	no	.	.
	6	33,000	no	.	.
	7	36,000	no	.	.

Survival Table

TPO at diagnosis +/-		N of Cumulative Events	N of Remaining Cases
Positive at onset	1	1	6
	2	2	5
	3	3	4
	4	4	3
	5	4	2
	6	4	1
	7	4	0

Means and Medians for Survival Time

	Mean[a]				Median	
			95% Confidence Interval			
TPO at diagnosis +/-	Estimate	Std. Error	Lower Bound	Upper Bound	Estimate	Std. Error
Negative at onset	32,871	1,100	30,715	35,027	.	.
Positive at onset	19,143	5,741	7,891	30,395	16,000	15,712
Overall	30,907	1,426	28,111	33,703	.	.

Means and Medians for Survival Time

	Median	
	95% Confidence Interval	
TPO at diagnosis +/-	Lower Bound	Upper Bound
Negative at onset	.	.
Positive at onset	,000	46,795
Overall	.	.

a. Estimation is limited to the largest survival time if it is censored.

Overall Comparisons

	Chi-Square	df	Sig.
Log Rank (Mantel-Cox)	9,429	1	,002

Test of equality of survival distributions for the different levels of TPO at diagnosis +/-.

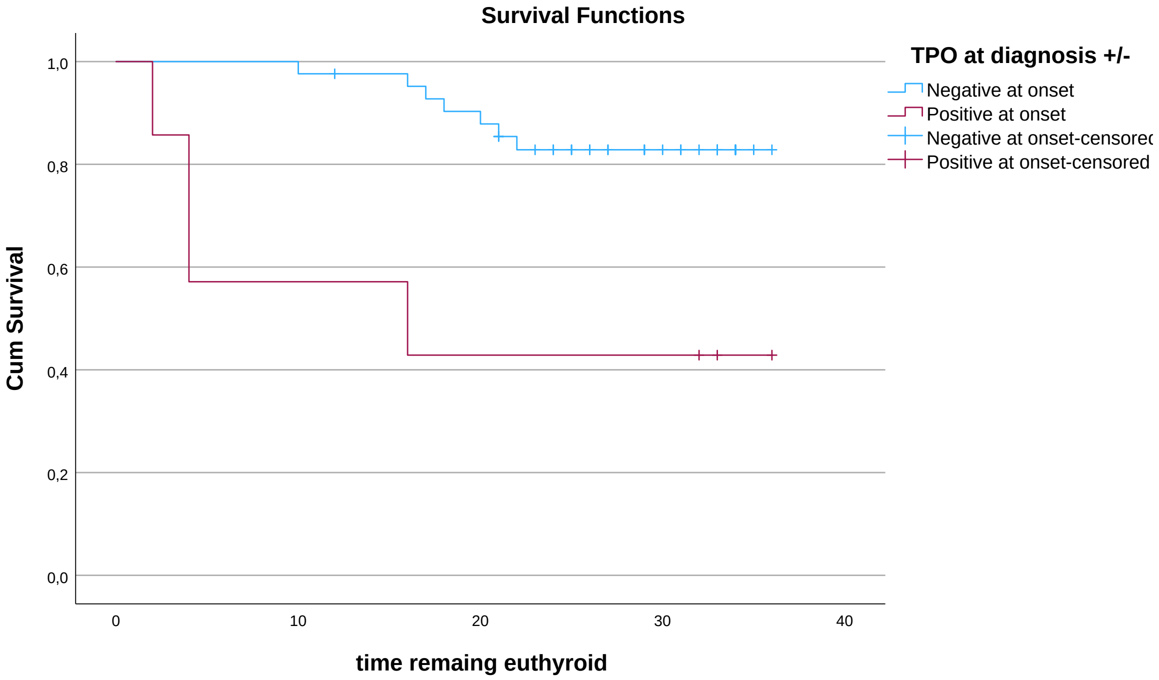

Kaplan-Meier : PTIENTS >= 18 YEARS AT DIABETES ONSET

Notes

Output Created		29-SEP-2025 14:04:06
Comments		
Input	Data	\\dspau.santpau.es\w\gcarreras\info\Escriptori\TIROIDES\listado AC 30-11-2023 angles.sav
	Active Dataset	DataSet1
	Filter	(seguimen >= 10) AND (edaddx >= 18) (FILTER)
	Weight	<none>
	Split File	<none>
	N of Rows in Working Data File	111
Missing Value Handling	Definition of Missing	User-defined missing values are treated as missing.
	Cases Used	Statistics are based on all cases with valid data for all variables in the analysis.
Syntax		KM Supervivència BY tpoqbis<br>/STATUS=disftir(1)<br>/PRINT TABLE MEAN<br>/PLOT SURVIVAL<br>/TEST LOGRANK<br>/COMPARE OVERALL POOLED.
Resources	Processor Time	00:00:00,23
	Elapsed Time	00:00:00,23

Case Processing Summary

TPO at diagnosis +/-	Total N	N of Events	Censored	
			N	Percent
Negative at onset	93	7	86	92,5%
Positive at onset	18	17	1	5,6%
Overall	111	24	87	78,4%

Survival Table

TPO at diagnosis +/-		Time	Status	Cumulative Proportion Surviving at the Time	
				Estimate	Std. Error
Negative at onset	1	10,000	si	,989	,011
	2	11,000	no	.	.
	3	12,000	si	.	.
	4	12,000	si	,968	,018
	5	14,000	no	.	.
	6	15,000	no	.	.
	7	17,000	si	,956	,021
	8	21,000	si	,945	,024
	9	22,000	si	,934	,026
	10	22,000	no	.	.
	11	22,000	no	.	.
	12	22,000	no	.	.
	13	24,000	no	.	.
	14	24,000	no	.	.
	15	25,000	no	.	.
	16	25,000	no	.	.
	17	25,000	no	.	.
	18	26,000	si	,922	,028
	19	26,000	no	.	.
	20	26,000	no	.	.
	21	26,000	no	.	.
	22	26,000	no	.	.
	23	26,000	no	.	.
	24	27,000	no	.	.
	25	27,000	no	.	.
	26	27,000	no	.	.
	27	27,000	no	.	.
	28	27,000	no	.	.
	29	28,000	no	.	.
	30	28,000	no	.	.
	31	28,000	no	.	.
	32	28,000	no	.	.
	33	28,000	no	.	.
	34	28,000	no	.	.
	35	29,000	no	.	.
	36	29,000	no	.	.
	37	29,000	no	.	.
	38	29,000	no	.	.
	39	29,000	no	.	.
	40	29,000	no	.	.
	41	29,000	no	.	.

Survival Table

TPO at diagnosis +/-		N of Cumulative Events	N of Remaining Cases
Negative at onset	1	1	92
	2	1	91
	3	2	90
	4	3	89
	5	3	88
	6	3	87
	7	4	86
	8	5	85
	9	6	84
	10	6	83
	11	6	82
	12	6	81
	13	6	80
	14	6	79
	15	6	78
	16	6	77
	17	6	76
	18	7	75
	19	7	74
	20	7	73
	21	7	72
	22	7	71
	23	7	70
	24	7	69
	25	7	68
	26	7	67
	27	7	66
	28	7	65
	29	7	64
	30	7	63
	31	7	62
	32	7	61
	33	7	60
	34	7	59
	35	7	58
	36	7	57
	37	7	56
	38	7	55
	39	7	54
	40	7	53
	41	7	52

Survival Table

TPO at diagnosis +/-		Time	Status	Cumulative Proportion Surviving at the Time	
				Estimate	Std. Error
	42	29,000	no	.	.
	43	30,000	no	.	.
	44	30,000	no	.	.
	45	30,000	no	.	.
	46	30,000	no	.	.
	47	30,000	no	.	.
	48	30,000	no	.	.
	49	30,000	no	.	.
	50	30,000	no	.	.
	51	30,000	no	.	.
	52	30,000	no	.	.
	53	31,000	no	.	.
	54	31,000	no	.	.
	55	31,000	no	.	.
	56	31,000	no	.	.
	57	31,000	no	.	.
	58	31,000	no	.	.
	59	31,000	no	.	.
	60	32,000	no	.	.
	61	32,000	no	.	.
	62	32,000	no	.	.
	63	32,000	no	.	.
	64	32,000	no	.	.
	65	32,000	no	.	.
	66	32,000	no	.	.
	67	32,000	no	.	.
	68	32,000	no	.	.
	69	32,000	no	.	.
	70	32,000	no	.	.
	71	33,000	no	.	.
	72	33,000	no	.	.
	73	33,000	no	.	.
	74	33,000	no	.	.
	75	33,000	no	.	.
	76	33,000	no	.	.
	77	33,000	no	.	.
	78	33,000	no	.	.
	79	33,000	no	.	.
	80	34,000	no	.	.
	81	34,000	no	.	.
	82	34,000	no	.	.

Survival Table

TPO at diagnosis +/-	N of Cumulative Events	N of Remaining Cases
42	7	51
43	7	50
44	7	49
45	7	48
46	7	47
47	7	46
48	7	45
49	7	44
50	7	43
51	7	42
52	7	41
53	7	40
54	7	39
55	7	38
56	7	37
57	7	36
58	7	35
59	7	34
60	7	33
61	7	32
62	7	31
63	7	30
64	7	29
65	7	28
66	7	27
67	7	26
68	7	25
69	7	24
70	7	23
71	7	22
72	7	21
73	7	20
74	7	19
75	7	18
76	7	17
77	7	16
78	7	15
79	7	14
80	7	13
81	7	12
82	7	11

Survival Table

TPO at diagnosis +/-		Time	Status	Cumulative Proportion Surviving at the Time	
				Estimate	Std. Error
	83	34,000	no	.	.
	84	34,000	no	.	.
	85	34,000	no	.	.
	86	34,000	no	.	.
	87	35,000	no	.	.
	88	35,000	no	.	.
	89	35,000	no	.	.
	90	35,000	no	.	.
	91	36,000	no	.	.
	92	37,000	no	.	.
	93	37,000	no	.	.
Positive at onset	1	,000	si	.	.
	2	,000	si	,889	,074
	3	1,000	si	,833	,088
	4	2,000	si	.	.
	5	2,000	si	,722	,106
	6	4,000	si	,667	,111
	7	5,000	si	.	.
	8	5,000	si	,556	,117
	9	6,000	si	,500	,118
	10	7,000	si	.	.
	11	7,000	si	.	.
	12	7,000	si	,333	,111
	13	9,000	si	,278	,106
	14	12,000	si	,222	,098
	15	15,000	si	,167	,088
	16	22,000	si	,111	,074
	17	25,000	si	,056	,054
	18	26,000	no	.	.

Survival Table

TPO at diagnosis +/-		N of Cumulative Events	N of Remaining Cases
	83	7	10
	84	7	9
	85	7	8
	86	7	7
	87	7	6
	88	7	5
	89	7	4
	90	7	3
	91	7	2
	92	7	1
	93	7	0
Positive at onset	1	1	17
	2	2	16
	3	3	15
	4	4	14
	5	5	13
	6	6	12
	7	7	11
	8	8	10
	9	9	9
	10	10	8
	11	11	7
	12	12	6
	13	13	5
	14	14	4
	15	15	3
	16	16	2
	17	17	1
	18	17	0

Means and Medians for Survival Time

TPO at diagnosis +/-	Mean[a]				Median	
			95% Confidence Interval			
	Estimate	Std. Error	Lower Bound	Upper Bound	Estimate	Std. Error
Negative at onset	35,464	,578	34,332	36,596	.	.
Positive at onset	8,611	1,891	4,905	12,317	6,000	1,061
Overall	31,188	1,102	29,028	33,349	.	.

Means and Medians for Survival Time

TPO at diagnosis +/-	Median 95% Confidence Interval Lower Bound	Upper Bound
Negative at onset	.	.
Positive at onset	3,921	8,079
Overall	.	.

a. Estimation is limited to the largest survival time if it is censored.

Overall Comparisons

	Chi-Square	df	Sig.
Log Rank (Mantel-Cox)	131,562	1	<,001

Test of equality of survival distributions for the different levels of TPO at diagnosis +/-.

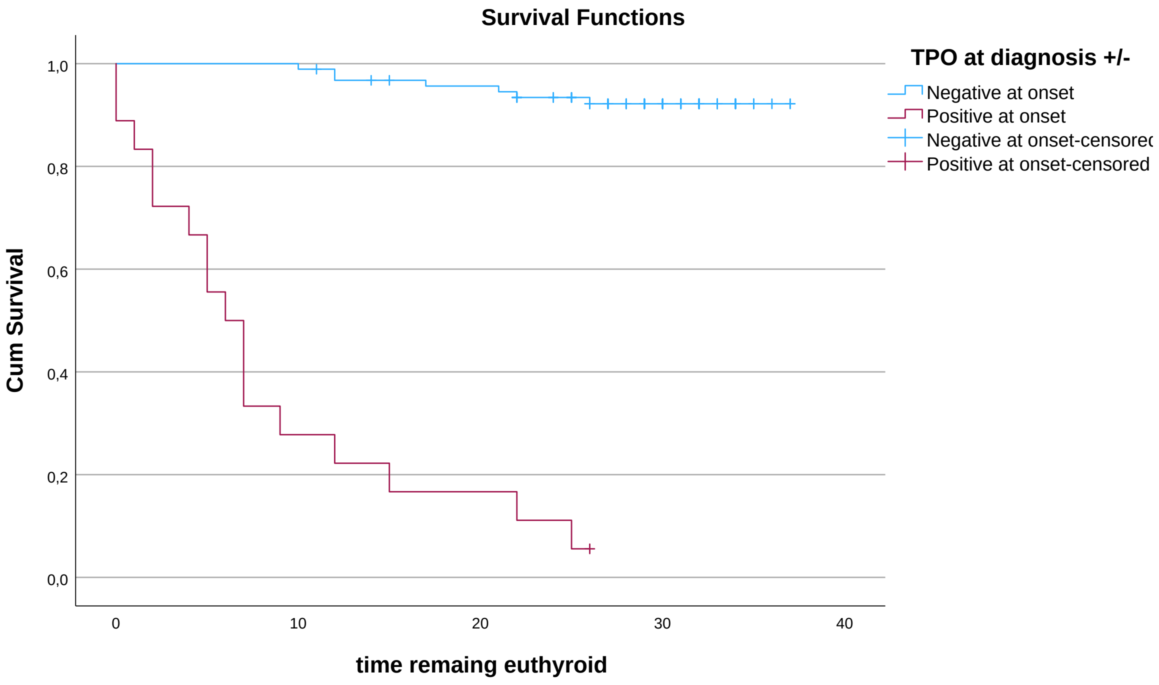

Supplement: Supplementary file 1 [file DataSheet1.pdf]
